# The Effect of Time-Restricted Eating Combined with Exercise on Body Composition and Metabolic Health: A Systematic Review and Meta-Analysis

**DOI:** 10.1016/j.advnut.2024.100262

**Published:** 2024-06-17

**Authors:** Zihan Dai, Kewen Wan, Masashi Miyashita, Robin Sze-tak Ho, Chen Zheng, Eric Tsz-chun Poon, Stephen Heung-sang Wong

**Affiliations:** 1 Department of Sports Science and Physical Education, The Chinese University of Hong Kong, Hong Kong, China; 2 Dr. Stephen Hui Research Centre for Physical Recreation and Wellness, Hong Kong Baptist University, Hong Kong, China; 3 Faculty of Sport Sciences, Waseda University, Saitama, Japan; 4 School of Sport, Exercise and Health Sciences, Loughborough University, Leicestershire, United Kingdom; 5 Department of Health and Physical Education, The Education University of Hong Kong, Hong Kong, China

**Keywords:** time-restricted eating, intermittent fasting, metabolic health, nutrition, exercise

## Abstract

**Background:**

Time-restricted eating (TRE) is increasingly popular, but its benefits in combination with exercise still need to be determined.

**Objectives:**

This systematic review and meta-analysis aimed to evaluate the efficacy of TRE combined with exercise compared with control diet with exercise in improving the body composition and metabolic health of adults.

**Methods:**

Five electronic databases were searched for relevant studies. Randomized controlled trials (RCTs) examining the effect of TRE combined with exercise on body composition and metabolic health in adults were included. All results in the meta-analysis are reported as mean difference (MD) with 95% confidence interval (CI). Study quality was assessed using the revised Cochrane Risk of Bias Tool and Grading of Recommendations Assessment, Development, and Evaluation assessment.

**Results:**

In total, 19 RCTs comprising 568 participants were included in this systematic review and meta-analysis. TRE combined with exercise likely reduced the participants’ body mass (MD: −1.86 kg; 95% CI: −2.75, −0.97 kg) and fat mass (MD: −1.52 kg; 95% CI: −2.07, −0.97 kg) when compared with the control diet with exercise. In terms of metabolic health, the TRE combined with exercise group likely reduced triglycerides (MD: −13.38 mg/dL, 95% CI: −21.22, −5.54 mg/dL) and may result in a reduction in low-density lipoprotein (MD: −8.52 mg/dL; 95% CI: −11.72, −5.33 mg/dL) and a large reduction in leptin (MD: −0.67 ng/mL; 95% CI: −1.02, −0.33 ng/mL). However, TRE plus exercise exhibited no additional benefit on the glucose profile, including fasting glucose and insulin, and other lipid profiles, including total cholesterol and high-density lipoprotein concentrations, compared with the control group.

**Conclusions:**

Combining TRE with exercise may be more effective in reducing body weight and fat mass and improving lipid profile than control diet with exercise. Implementing this approach may benefit individuals aiming to achieve weight loss and enhance their metabolic well-being.

This study was registered in PROSPERO as CRD42022353834.

**Statement of Significance**

This systematic review and meta-analysis provided evidence supporting the effectiveness of combining time-restricted eating (TRE) with exercise in reducing body weight and fat mass, as well as improving lipid profiles. These findings have significant implications for healthcare practitioners and public health professionals, offering valuable insights into the combined effects of TRE and exercise; however, further research is needed to compare effects of different exercise modalities and explore the combined intervention in distinct population groups separately.

## Introduction

A modern lifestyle characterized by the round-the-clock availability of high-calorie foods and insufficient regular physical activity accompanied by periods of prolonged sitting significantly perturbs the circadian clock. It increases susceptibility to metabolic diseases [1]. WHO recommends avoiding an unhealthy diet and exercising regularly to prevent and reduce major risk factors for noncommunicable diseases [2]. Intermittent fasting (IF) has emerged as a novel approach beyond simple calorie restriction to reduce body weight and improve metabolic health [3]. Time-restricted eating (TRE) is a form of IF that has emerged as a popular dietary strategy in recent years and involves confining the eating window to a specified number of hours per day and fasting with zero-calorie beverages for the remaining hours of the day [4]. Notably, TRE has grown prominently as a creative and workable treatment for obesity and metabolic diseases, because it eliminates the need to track caloric intake or calorie count during the eating window, making it a convenient and straightforward approach [5]. Emerging evidence suggests that modifying meal timings can prevent and manage various lifestyle-related disease states and affect a wide variety of physiological functions [6], including those linked to the circadian clock, athletic performance, skeletal muscle insulin sensitivity, and whole-body metabolic health [7].

Exercise is a well-known component of a healthy lifestyle [8], conferring benefits such as weight management, enhanced cardiovascular well-being, increased energy expenditure, improved mood, and decreased susceptibility to chronic diseases [9]. Previous meta-analyses have consolidated the benefits of integrating exercise and dietary interventions, highlighting that the combined approach surpasses individual interventions in optimizing overall effects and harnessing potential health benefits [10]. Several human trials [11,12] have investigated the metabolic benefits of combining TRE with exercise. In a randomized controlled trial (RCT), Haganes et al. [11] found that the combined approach of TRE and exercise resulted in a reduction in glycated hemoglobin (HbA1c) concentrations and visceral fat compared with the individual exercise or TRE groups, as well as the control group, in overweight/obese populations. Another RCT conducted by Moro et al. [12] found that TRE combined with exercise effectively reduced inflammatory markers and risk factors related to cardiovascular and metabolic diseases in healthy adults. Exercise and TRE have beneficial effects on almost all organ systems through overlapping mechanisms [13], such as the improvement of insulin sensitivity [14,15], autophagy activation [16,17], anti-inflammatory effects [18,19], and gut microbiota modulation [20,21]. Therefore, combined TRE and exercise may be an effective and practical lifestyle alternative for improving overall metabolic health.

A recent meta-analysis examined the effects of IF, including TRE, and other types such as alternate-day fasting and the 5:2 diet, combined with exercise. The findings revealed that IF plus exercise led to improvements in various cardiometabolic outcomes, including body weight, blood pressure, and lipid profile, compared with a control diet plus exercise [22]. However, previous meta-analyses have not comprehensively analyzed the additive effects of TRE plus exercise compared with a control diet plus exercise on body composition and multiple metabolic variables. Therefore, the purpose of this systematic review and meta-analysis was to consolidate and quantify the available data on the combination of TRE and exercise, as well as assess its efficacy in improving body composition and metabolic health compared with following a control diet with exercise. By understanding the combined effects of TRE and exercise, we can potentially enhance the development of more effective lifestyle interventions and provide personalized recommendations for individuals aiming to optimize their overall health and well-being.

## Methods

### Registration

This systematic review and meta-analysis was registered at PROSPERO (registration number CRD42022353834) and performed following the PRISMA statement guidelines and the Cochrane Handbook of Systematic Reviews of Interventions [23].

### Search strategy

All literature investigating the combined effect of TRE and exercise on body composition and metabolic health were searched and obtained utilizing PubMed, Embase, SPORTDiscus, Web of Science, and Cochrane Central Register of Controlled Trials from inception to February 2024. The search strategy included various combinations of the keywords and MeSH terms: (time-restricted feeding or time-restricted eating or intermittent fasting or time-restricted fasting or time-restricted diet or time-restricted meal) AND (exercise or physical activity or physical exercise or training or fitness or running or cycling). A detailed search strategy is presented in Supplementary Table 1. These searches were limited to human studies and full text, the papers accepted were in English language only, and no restriction was applied on the publication date. Additional studies were identified by searching the reference lists of the studies that were obtained by our systematic search.

### Eligibility criteria and study selection

**FIGURE 1:**
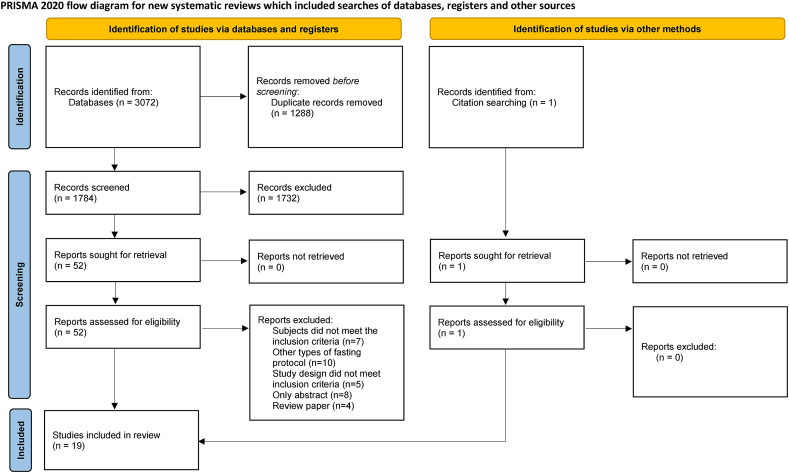
Flowchart of study selection.

The study selection process is shown in Figure 1. Search results retrieved by the search strategy were imported into Endnote 20. Two authors (ZD and KW), working together, completed the initial screening of records according to the defined criteria. Once the titles and abstracts were screened, 2 authors reviewed the full texts independently and discussed discrepancies, and any disagreements between the 2 authors were resolved through discussion with a third author (RH) until a consensus was reached. The following defined inclusion criteria according to the population, intervention, comparison, outcome framework were used to select studies eligible for this systematic review and meta-analysis: *1*) a population of healthy individuals aged ≥18 y with normal weight, overweight, or obesity; *2*) RCTs or randomized crossover studies with intervention of TRE combined with all types of exercise; *3*) control diet (control diet describes a diet not following the TRE pattern; different studies used various terms including habitual diet and usual diet, which describe eating habits that individuals consistently follow over time; non-TRE diet, which refers to an unrestricted eating window diet; normal dietary pattern, which describes a dietary pattern that is considered typical or standard within a given population) with the same exercise program; and *4*) outcome measures included body composition (body mass, fat mass, and fat-free mass) and parameters related to lipid profiles (total cholesterol [TC], LDL cholesterol, HDL cholesterol, and triglycerides [TG]), fasting glucose and insulin concentrations, inflammatory cytokines and adipokines (insulin-like growth factor 1 [IGF-1], IL-6, TNF-α, leptin, and adiponectin].

### Data extraction

Extraction of data from included studies was performed by a single author (ZD) into an electronic spreadsheet (Excel, 2023) according to the following study characteristics: study information (first author, publication year, characteristics of the participants, and study design); intervention duration; TRE eating window and calorie intake; exercise type; and outcomes and main findings. Forest plots were generated by conducting meta-analyses using the postintervention means and SDs or mean differences (MDs) and their corresponding SDs for each outcome. All the outcomes were transferred to the same unit, such as body weight (kilogram), fat mass (kilogram), LDL (milligrams per deciliter), TG (milligrams per deciliter), and fasting glucose (milligrams per deciliter). In articles in which the means and SDs were not provided, we contacted the authors for the relevant data. When it was impossible to obtain adequate data due to communication failure, data were extracted from graphical representations using WebPlotDigitizer software. A second author (KW) checked all data extracted for accuracy.

### Risk of bias assessment

The revised Cochrane Risk of Bias tool (RoB 2) [24] and RoB 2 additional considerations for crossover trials [25] were independently used by 2 authors (ZD and KW) to assess the risk of bias, and any disagreements were resolved by discussion with the other authors. The following aspects were evaluated for the quality of the RCTs: *1*) bias arising from the randomization process; *2*) bias because of deviations from the intended interventions; *3*) bias because of missing outcome data; *4*) bias in the measurement of the outcome; and *5*) bias in the selection of the reported result. The crossover trial assessed 6 domains: randomization, period and carryover effects, deviation from the intended intervention, missing outcome data, measurement of the outcome, and selection of reported results. Moreover, each study’s overall risk of bias was determined as low risk, some concerns, or high risk.

### Certainty of evidence

The Grading of Recommendation Assessment, Development, and Evaluation (GRADE) assessment was used to assess the overall quality of evidence. The overall quality was assessed on the following domains: risk of bias, consistency of results across studies, directness and precision of results, and the likelihood of publication bias [26]. GRADE assessments were conducted for all the outcomes included in the meta-analysis. All authors examined any disagreements before reaching a consensus.

### Data synthesis and analysis

One author (ZD) performed data synthesis and analysis, and meta-analyses were completed utilizing Review Manager software (RevMan Version 5.4, Cochrane Collaboration). These analyses involved calculating MDs along with 95% confidence intervals (CIs) to assess outcomes. The MD is a standard statistic that measures the absolute difference between the mean values in 2 groups of a randomized trial. The calculations were performed using random-effects models, considering the possibility of heterogeneity in clinical or methodological factors that could have influenced the outcomes. Effect sizes were computed to assess and compare the effects of TRE combined with exercise with the control diet with exercise for the outcomes. Heterogeneity was evaluated utilizing the *I*^2^ statistic, with a significance level set at *P* < 0.05. Analyses with *I*^2^ > 50% were explored using sensitivity analyses, excluding one study at a time, to verify whether any study was responsible for heterogeneity. Subgroup analyses were performed as follows: *1*) TRE calorie intake: ad libitum TRE (unrestricted energy intake within the eating window) and non-ad libitum TRE (restricted energy intake within the eating window); and *2*) intervention duration (moderate-term interventions >4 wk, or short-term interventions ≤4 wk). Publication bias was examined with a funnel plot and Egger’s test if ≥10 studies were included in the meta-analysis. An α value equal to 5% was adopted for all analyses. The analyses were conducted using the metal package of the software Stata v 13.0 (StataCorp).

## Results

### Study selection

A total of 3072 papers were initially identified from database searches, and after removing 1288 duplicates, title and abstract screening excluded 1732 studies. Of the remaining 52 full-text papers assessed for eligibility, 34 were excluded (7 had inappropriate subjects, 10 used other types of fasting protocol, 5 had inappropriate study design, 8 were only abstracts, and 4 were review papers). One article was added through citation searching. Nineteen final articles were identified to be eligible for inclusion in the review and meta-analysis [11,12,[27], [28], [29], [30], [31], [32], [33], [34], [35], [36], [37], [38], [39], [40], [41], [42], [43]]. The PRISMA diagram of the selection process is detailed in Figure 1.

### Study characteristics

Table 1 [11,12,[27], [28], [29], [30], [31], [32], [33], [34], [35], [36], [37], [38], [39], [40], [41], [42], [43]] summarizes the general characteristics of the 19 RCTs included in the meta-analysis. The articles by Richardson et al. [39] and Tovar et al. [43] are from the same study. However, the former reports the metabolic biomarkers, whereas the latter reports the body composition and performance as outcomes. The studies included a range of 12–131 participants (*n* = 568). Intervention periods ranged from 4 wk to 12 mo; 7 studies were 4 wk [[29], [30], [31],^37^,^39^,^40^,^43^], 1 study was 7 wk [11], 10 studies were 8 wk [27,28,[32], [33], [34], [35], [36], 38,41,42], and 1 study was 12 mo [12]. The age of participants ranged from 18 to 62 y. Eleven studies only included male participants [12,[28], [29], [30], [31],^36^,^37^,[39], [40], [41],^43^], 6 studies only included female participants [11,27,[33], [34], [35],^42^], and 2 studies included both male and female participants [32,38]. Thirteen of the studies were performed in active, healthy participants [12,[28], [29], [30], [31],[35], [36], [37],[39], [40], [41], [42], [43]], whereas 6 studies included adults with overweight/obesity [11,27,[32], [33], [34],^38^]. Eight studies conducted TRE using the ad libitum approach [11,[28], [29], [30], [31], [32],^34^,^41^], whereas the other 11 used the non-ad libitum approach [12,27,33,[35], [36], [37], [38], [39], [40],^42^,^43^].

**TABLE 1:** Characteristics of the included studies

Author	Participants (health status, *N*, sex, age)	Study design	Study duration	TRE strategy	Training protocol	Intervention comparison	Main outcomes	Main findings
Batitucci et al. 2022 [27]	Women with obesity, N = 36; 32.2 ± 4.4 y	Randomized controlled trial	8 wk	6/18, self-selected eating window Non-ad libitum: calorie restriction	High-intensity interval training: 25 min each session, with 4 min of initial warm-up, 18 min for the main part, and 3 min of relaxation	TRE+EX CD+EX	Body composition including weight, BMI, WC, HC, RMR, lipid oxidation Food consumption including calories and macronutrients	TRE+EX vs. CD+EX: ° Body weight↓ ° BMI↓ TRE+EX and CD+EX: ° Body fat mass ↓ ° Fat-free mass ↑
Brady et al. 2021 [28]	Male middle- and long-distance runners, N = 17; 36.4 ± 7.4 y	Randomized controlled trial	8 wk	8/16, 12:00–20:00 Ad libitum	Cycling training for a minimum of 28 sessions during the intervention	TRE+EX HD+EX	Body composition including body mass, fat mass, and fat-free mass Daily energy intake Markers of metabolic health including glucose, insulin, triglycerides, and HOMA-IR	TRE+EX vs. HD+EX: ° Body mass ↓
Correia et al. 2021 [29]	Healthy male physical education students, N = 12; 22.4 ± 2.8 y	Randomized crossover trial	4 wk	8/16, 13:00–21:00 Ad libitum	Power sports training ≥3 times/wk	TRE+EX non-TRE+EX	Body composition including body mass, fat mass, fat-free mass Energy intake and macronutrient distribution	TRE+EX and non-TRE+EX: ° Fat-free mass ↑
Correia et al. 2023 [30]	Healthy, trained male physical education students, N = 18; 23.7 ± 2.6 y	Randomized crossover trial	4 wk	8/16, 13:00–21:00 Ad libitum	4 sets of maximum repetitions at 85% 1-RM in 5 dynamic exercises, 3 times/wk	TRE+EX non-TRE+EX	Body composition including body mass, fat mass, fat-free mass, skeletal muscle Dietary intake	TRE+EX and non-TRE+EX: ° Fat mass ↓
Correia et al. 2024 [31]	Trained male physical education students, N = 15; 23.7 ± 2.6 y	Randomized crossover trial	4 wk	8/16, 13:00–21:00 Ad libitum	3 outdoor runs ×10 km/wk within the heavy domain + 3 running bouts ×1000 m	TRE+EX HD+EX	Body composition including body mass, fat mass, fat mass%, fat-free mass, skeletal muscle Markers of metabolic health including triglycerides, total cholesterol, HDL, LDL, non-HDL, Fasting blood glucose Energy intake	TRE+EX vs. HD+EX: ° NS
Haganes et al. 2022 [11]	Women with overweight /obesity, N = 131; 36.2 ± 6.2 y	Randomized controlled trial	7 wk	A self-selected eating window of ≤10 h/d Ad libitum	2 weekly sessions: 4 × 4-min work bouts at 90%–95% HRmax, separated by 3 min moderate-intensity recovery Third session: 10 × 1-min work bouts at ≥90% HRmax separated by 1 min low-intensity recovery	TRE+EX HD+EX	Oral glucose tolerance test outcomes Glycemic control including HbA1c, glucose, insulin, HOMA2-IR Body composition including weight, fat mass, muscle mass, visceral fat Cardiometabolic markers including glucose, lipid, leptin, and adiponectin Dietary intake	TRE+EX: ° HbA1c ↓ TRE+EX vs. HD+EX: ° Body weight ↓ ° Fat mass ↓ ° Visceral fat area ↓
Kotarsky et al. 2021 [32]	Overweight or obese adults, N = 21 (3 males); 44 ± 7 y	Randomized controlled trial	8 wk	8/16, 12:00–20:00 Ad libitum	Resistance training: 3 different workouts, performed on nonconsecutive days, each week for 8 wk Aerobic training: Complete the goal of a total of 300 min of moderate or 150 min of vigorous physical activity per week	TRE+EX ND+EX	Body composition including body mass, BMI, HC, WC, lean mass, fat mass, BMC, BMD Resting cardiometabolic including insulin, hsCRP, HbA1c, HDL, estradiol, progesterone, testosterone, DEHAS, cortisol	TRE+EX and ND+EX: ° Lean mass ↑ TRE+EX vs. ND+EX: ° Body mass ↓ ° BMI ↓ ° Fat mass ↓
Lin et al. 2022 [33]	Middle-aged women, N = 63; TRE+EX: 50.1 ± 7.5 y non-TRE+EX: 54.2 ± 7.9 y	Randomized controlled trial	8 wk	8/16, either 10:00–18:00 or 12:00–20:00 Non-ad libitum: calorie restriction	8 30-min exercise sessions	TRE+EX non-TRE+EX	Body composition including body weight, BMI, WC, BF%, fat-free mass, waist-to-hip ratio Cardiometabolic risk factors including fasting glucose, TC, HDL, LDL, triacylglycerols, SBP, DBP, fasting insulin, HOMA-IR	TRE+EX vs. non-TRE+EX: ° BMI ↓ ° Body weight ↓ ° DBP ↓
Liu et al. 2023 [34]	Female college students with hidden obesity, N = 77; 18–22 y	Randomized controlled trial	8 wk	8/16, 10:00–18:00 Ad libitum	Walking exercise, achieve 11,000–12,499 steps per day	TRE+EX UD+EX	Body composition including weight, BMI, BF%, ATM, LTM, TBMD Lipid profile including TC, TG, HDL, LDL; DBP, SBP	TRE+EX vs. UD+EX: ° NS
Martínez-Rodríguez et al. 2021 [35]	Active normal-weight women, N = 14; 27 ± 6 y	Randomized crossover design	8 wk	Not eat in <14 h of the day before, and consume breakfast as soon as possible after waking and continue to eat following the diet intervals throughout the remainder of the day Non-ad libitum: isocaloric eating	3 × 10 repetitions of 30 s of aerobic exercises interspersed by 30 s of rest and 3 times/wk (training session interval 48 h)	TRE+EX UD+EX	Body composition including fat mass, muscle mass, residual mass, body mass, skinfolds	TRE+EX vs. UD+EX: ° Fat mass ↓
Moro et al. 2016 [36]	Resistance-trained males, N = 34; TRE+EX: 29.94 ± 4.07 y ND+EX: 28.47 ± 3.48 y	Randomized controlled trial	8 wk	8/16, 3 meals consumed at 13:00, 16:00, and 20:00 Non-ad libitum: isocaloric eating	3 different weekly sessions (resistance training)	TRE+EX ND+EX	Diet composition Macronutrients distribution Body composition including fat-free mass, fat mass, body weight Metabolic risk factors including adiponectin, leptin, IL-6, TFN- α, insulin, TSH, T3, glucose, TC, cortisol, HDL, LDL, TG, REE, RR, IL-1β, testosterone total, IGF-1	TRE+EX vs. ND+EX: ° Fat mass ↓ ° Testosterone ↓ ° IGF-1 ↓ ° TG ↓ ° Adiponectin ↑ ° Leptin ↓ ° IL-1β ↓
Moro et al. 2020 [37]	Elite under-23 male cyclists, N = 16; 19.3 ± 0.1 y	Randomized controlled trial	4 wk	8/16, breakfast (10:00–11:00); lunch (13:00–14:00); dinner (18:00–19:00) Non-ad libitum: Isocaloric eating	500 ± 50 km/wk divided into 6 sessions per week that took place within the feeding time window (10:00–18:00)	TRE+EX ND+EX	Diet composition Macronutrient distribution Body composition including body mass, fat-free mass, and fat mass Blood biochemistry results include complete blood count, white blood cells, glucose, creatinine, creatine kinase, TC, TG, iron, ferritin, transferrin, CRP, TSH, testosterone, SHBG, cortisol, insulin, IL-6, adiponectin, TNF-α, IGF-1	TRE+EX vs. ND+EX: ° Total body mass ↓ ° Fat mass ↓ ° Free testosterone ↓ ° IGF-1 ↓
Moro et al. 2021 [12]	Healthy, regular resistance training males, N = 20; TRE+EX: 29.94 ± 4.07 y ND+EX: 28.47 ± 3.48 y	Randomized controlled trial	12 mo	8/16, 3 meals consumed at 13:00, 16:00, and 20:00 Non-ad libitum: isocaloric eating	3 different weekly sessions (resistance training)	TRE+EX ND+EX	Diet composition Macronutrient distribution Body composition including body weight, fat-free mass, fat mass, REE, RR Blood parameters including testosterone, IGF-1, TSH, T3, adiponectin, leptin, glucose, insulin, HOMA-IR, TC, HDL, LDL, TG, IL-6, IL-1β, TNF-α	TRE+EX v.s. ND+EX: ° Body mass, fat mass ↓ ° IGF-1 ↓ ° Testosterone ↓ ° IL-6, IL-β, TNF-α ↓ ° Fasting glucose, insulin, HOMA-IR ↓ ° Triglycerides ↓ ° HDL cholesterol ↑ ° Adiponectin ↑ ° Leptin ↓
Peeke et al. 2021 [38]	Participants with obesity, N = 60 (88% women); 44 ± 11 y	Randomizedcomparator-controlled, clinical trial	8 wk	10/14: the group consisted of a 14-h metabolic fast that began after dinner (between 17:00 and 20:00) and ended with the consumption of breakfast 14 h later Non-ad libitum: calorie restriction	Exercise program: walking exercise count to between 7000–10,000 steps/d	TRE+EX ND+EX	Body weight and fasting blood glucose	TRE+EX vs. ND+EX: ° Fasting blood glucose ↓ ° Body weight ↓
Richardson et al. 2023 [39]	Male long-distance runners, N = 15; 28. 7 ± 5.2 y	Randomized crossover trial	4 wk	8/16, not the same period for all participants Non-ad libitum: isocaloric eating	A personalized 4-wk training routine ≥32 km·week-1 based on their established training methods	TRE+EX ND+EX	Dietary intake Body composition including body bass, fat mass, fat-free mass, BF%, android/gynoid ratio, BMD REE including REE, RER Insulin resistance and sensitivity including glucose, insulin, HOMA-IR, QUICKI Blood pressure Circulating lipids and lipoproteins	TRE+EX vs. ND+EX: ° Whole-body fat mass ↓ ° Leg fat mass ↓ ° BF% ↓
Stratton et al. 2020 [40]	Recreationally active males, N = 26; TRE+EX: 22.9 ± 3.6 y ND+EX: 22.5 ± 2.2 y	Randomized controlled trial	4 wk	8/16, 12:00–20:00 or 13:00–21:00 Non-ad libitum: calorie restriction	Full body sessions performed 3 times/wk (resistance training)	TRE+EX ND+EX	Dietary intake Body composition including body mass, fat mass, BF%, fat-free mass Muscle morphology REE Blood biomarkers including testosterone, cortisol, adiponectin, leptin, ghrelin	TRE+EX v.s. ND+EX: NS TRE+EX & ND+EX: ° Body mass ↓ ° Fat mass ↓ ° Body fat% ↓ ° Testosterone ↓ ° Adiponectin ↓ ° REE ↓
Tinsley et al. 2017 [41]	Young recreationally active males, N = 18; TRE+EX: 22.9 ± 4.1 y ND+EX: 22.0 ± 2.4 y	Randomized controlled trial	8 wk	Consume all calories in any 4-h window between 16:00 and 24:00 and no restriction of food intake on exercise days Ad libitum	Nonconsecutive workouts 3 d/wk (resistance training)	TRE+EX ND+EX	Dietary intake Body composition including body weight, lean soft tissue, fat mass, BF%	TRE+EX vs. ND+EX: ° NS
Tinsley et al. 2019 [42]	Healthy, active females, N = 40; 18–30 y	Randomized controlled trial	8 wk	8/16, not the same period for all participants Non-ad libitum: isocaloric eating	RT sessions were completed on 3 nonconsecutive days each week, and 2 different upper- and lower-body sessions were alternated	TRE+EX CD+EX	Nutrient intake Body composition including body mass, fat mass, fat-free mass, BF%, REE, RQ Blood variables including glucose, cholesterol, HDL, TG, VLDL, insulin, LDL Vascular assessments including blood pressure, heart rate, pulse wave velocity	TRE+EX v.s. CD+EX: ° Fat mass ↓ TRE+EX & CD+EX: ° Lean mass ↑
Tovar et al. 2021 [43]	Healthy, endurance-trained male runners, N = 15; 28.7 ± 5.2 y	Randomized crossover trial	4 wk	8/16, not the same period for all participants Non-ad libitum: isocaloric eating	A personalized 4-wk training routine ≥32 km·week-1 based on their established training methods	TRE+EX ND+EX	Body composition including body mass, lean mass, fat mass, BF%	TRE+EX vs. ND+EX: ° Fat mass ↓ ° BF% ↓

Abbreviations: 1-RM, one-repetition maximal; ATM, adipose tissue mass; BF%, body fat percentage; BMC, bone mineral content; BMD, bone mineral density; BMI, body mass index; CD, control diet; CRP, C-reactive protein; DBP, diastolic blood pressure; DEHAS, dehydroepiandrosterone sulfate; HbA1c, glycated hemoglobin; HC, hip circumference; HD, habitual diet; HDL, high-density lipoprotein cholesterol; HOMA-IR, homeostatic model assessment for insulin resistance; HRmax, maximal heart rate; hsCRP, high-sensitivity C-reactive protein; IGF-1, insulin-like growth factor 1; IL, interleukin; LDL, low-density lipoprotein cholesterol; LTM, lean tissue mass; non-HDL, total cholesterol minus high-density lipoprotein cholesterol; non-TRE, non–time-restricted eating; ND, normal diet; NS, not significant; QUICKI, quantitative insulin sensitivity check index; REE, resting energy expenditure; RER/RR, respiratory exchange ratio; RMR, resting metabolic rate; RQ, respiratory quotient; RT, resistance training; SBP, systolic blood pressure; SHBG, sex hormone-blinding globulin; T3, triiodothyronine; TBMD, total bone mineral density; TC, total cholesterol; TG, triglycerides; TNF-α, tumor necrosis factor alpha; TRE+EX, time-restricted eating combined with exercise; TSH, thyroid-stimulating hormone; UD, usual diet; VLDL, very low-density lipoprotein cholesterol; WC, waist circumference.

### Meta-analysis

#### Effects on body composition

**FIGURE 2:**
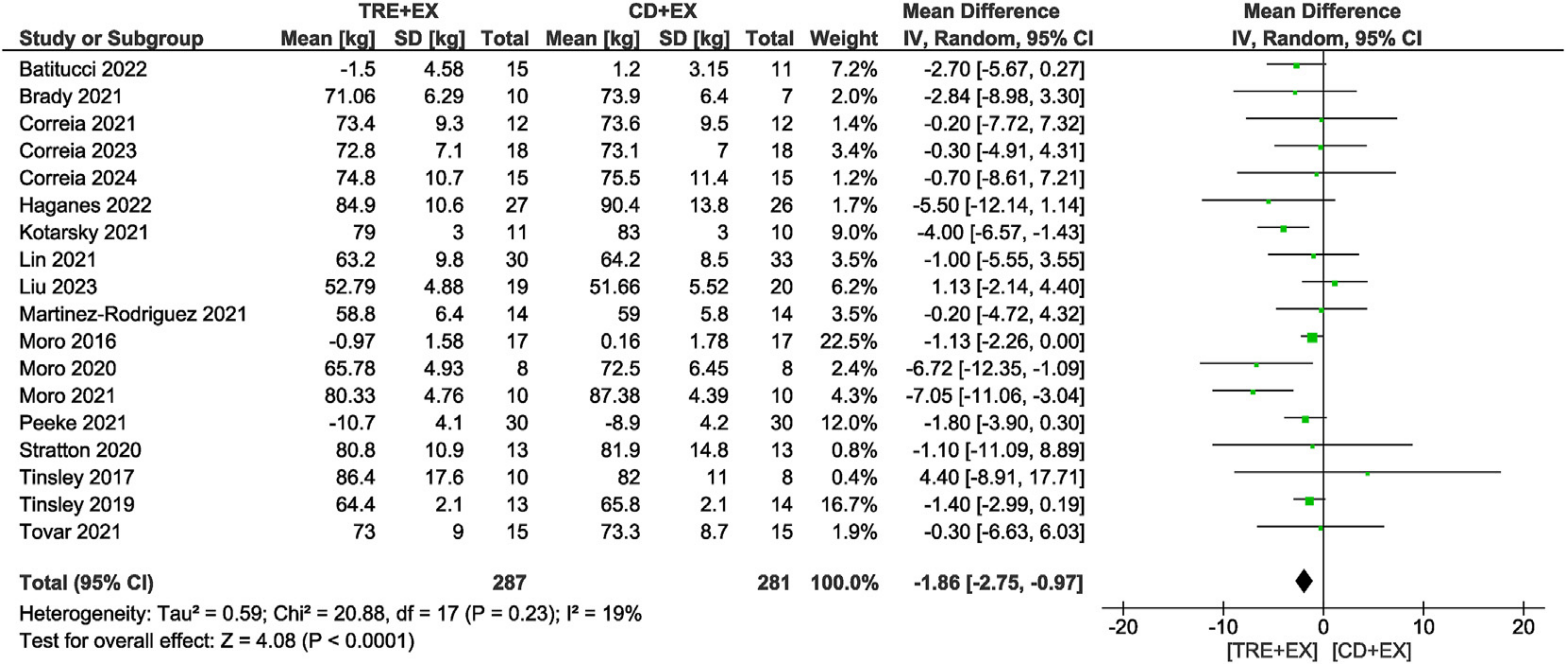
Forest plot of the effects of TRE + EX compared with CD + EX on body mass. CD, control diet; CI, confidence interval; EX, exercise; IV, inverse variance; SD, standard deviation; TRE, time-restricted eating.

**FIGURE 3:**
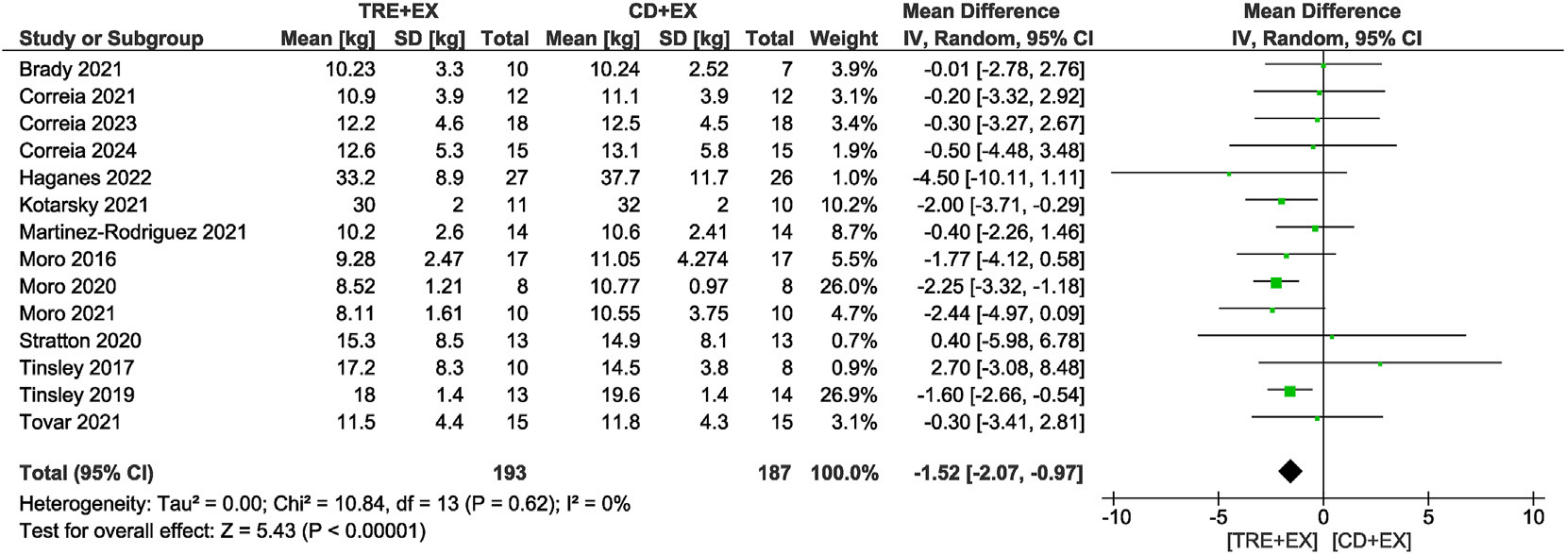
Forest plot of the effects of TRE + EX compared with CD + EX on fat mass. CD, control diet; CI, confidence interval; EX, exercise; IV, inverse variance; SD, standard deviation; TRE, time-restricted eating.

**FIGURE 4:**
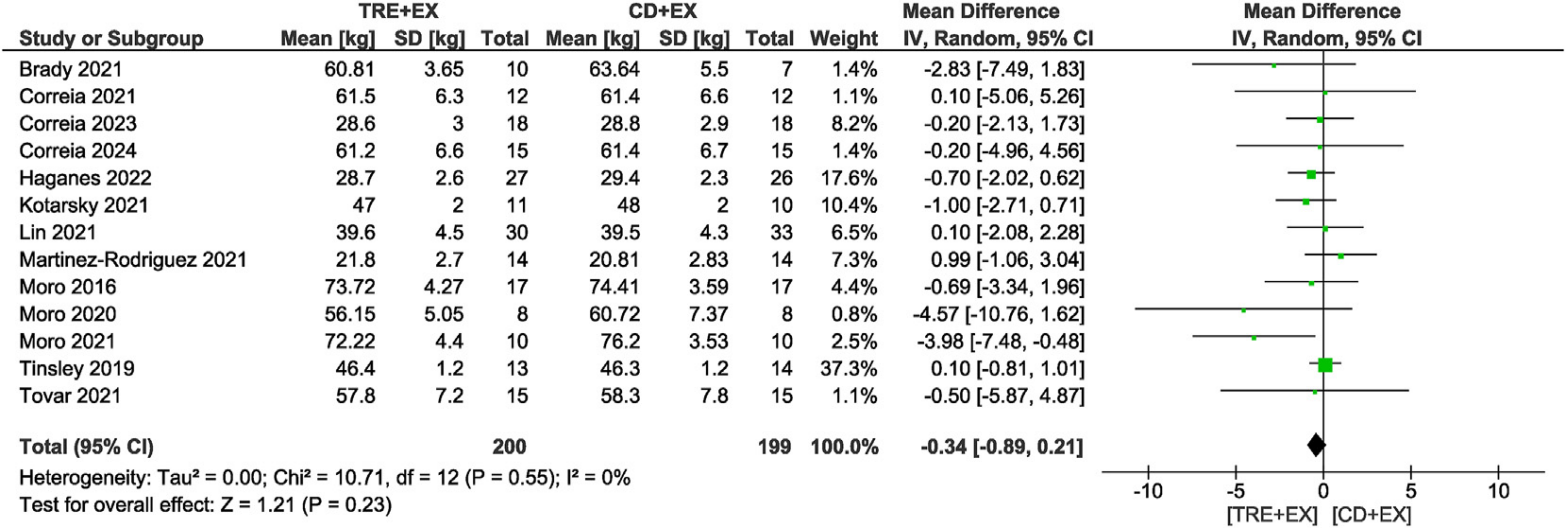
Forest plot of the effects of TRE + EX compared with CD + EX on fat-free mass. CD, control diet; CI, confidence interval; EX, exercise; IV, inverse variance; SD, standard deviation; TRE, time-restricted eating.

Eighteen studies [11,12,[27], [28], [29], [30], [31], [32], [33], [34], [35], [36], [37], [38],[40], [41], [42], [43]], including 568 participants, analyzed body mass as an outcome. Participants assigned to the combined strategy likely reduced body mass (MD: −1.86 kg; 95% CI: −2.75, −0.97 kg; *P* < 0.01; *I*^2^ = 19%) compared with the control group with moderate certainty of evidence (Figure 2). Subgroup analyses revealed that only non-ad libitum TRE probably decreased body mass compared with the control group (MD: −1.91 kg; 95% CI: −2.97, −0.86 kg; *P* < 0.01; *I*^2^ = 26%). In addition, subgroup analyses revealed reductions in body mass only for moderate-term interventions of >4 wk (MD: −1.93 kg; 95% CI: −2.99, −0.87 kg; *P* < 0.01; *I*^2^ = 35%). In terms of fat mass, the combined strategy likely resulted in a reduction (MD: −1.52 kg; 95% CI: −2.07, −0.97 kg; *P* < 0.01; *I*^2^ = 0%) when compared with the control diet plus exercise group with moderate certainty of evidence in 14 studies [11,12,[28], [29], [30], [31], [32],[35], [36], [37],[40], [41], [42], [43]] involving 380 participants (Figure 3). With the same trend as body mass, subgroup analyses revealed that only non-ad libitum TRE likely decreased fat mass compared with the control group (MD: −1.68 kg; 95% CI: −2.31, −1.05 kg; *P* < 0.01; *I*^2^ = 0%). The subgroup based on intervention duration showed that both durations likely showed similar reductions in fat mass. When considering fat-free mass, no differences were observed between the combined strategy and the control group (MD: −0.34 kg; 95% CI: −0.89, 0.21 kg; *P* = 0.23; *I*^2^ = 0%) with low certainty of evidence in 13 studies [11,12,[28], [29], [30], [31], [32], [33],[35], [36], [37],^42^,^43^] involving 399 participants (Figure 4). This result was consistent in subgroup analyses.

#### Effects on fasting glucose and insulin

**FIGURE 5:**
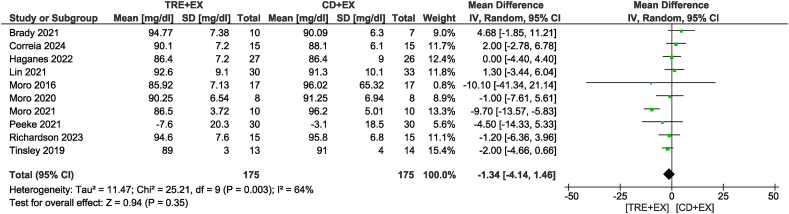
Forest plot of the effects of TRE + EX compared with CD + EX on fasting glucose. CD, control diet; CI, confidence interval; EX, exercise; IV, inverse variance; SD, standard deviation; TRE, time-restricted eating.

**FIGURE 6:**
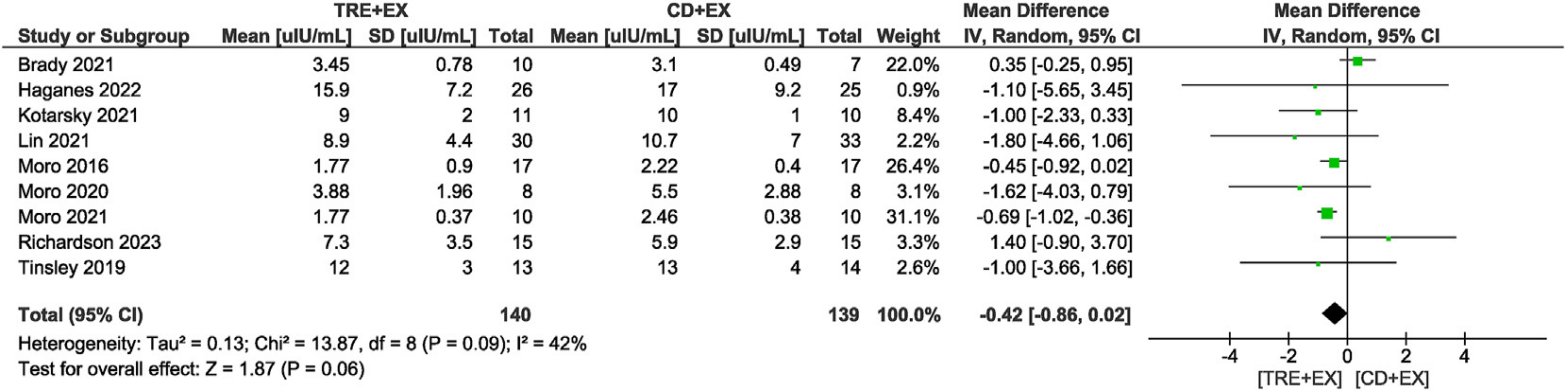
Forest plot of the effects of TRE + EX compared with CD + EX on fasting insulin. CD, control diet; CI, confidence interval; EX, exercise; IV, inverse variance; SD, standard deviation; TRE, time-restricted eating.

In the analysis of fasting glucose concentrations from 10 studies [11,12,28,31,33,[36], [37], [38], [39],^42^] involving 350 participants (Figure 5), there was no significant difference between the combined strategy and the control group (MD: −1.34 mg/dL; 95% CI: −4.14, 1.46 mg/dL; *P* = 0.35; *I*^2^ = 64%), with low certainty of evidence. After sensitivity analysis, we found that removing the study by Moro et al. [12] did not alter statistical significance but reduced heterogeneity (MD: −0.41 mg/dL; 95% CI: −2.04, 1.22 mg/dL; *P* = 0.62; *I*^2^ = 0%). Subgroup analysis also did not reveal any significant differences. Nine studies [11,12,28,32,33,36,37,39,42] analyzed insulin as an outcome, including 279 participants with low certainty of evidence (Figure 6). The combined strategy did not show differences in insulin concentrations compared with the control group (MD: −0.42 μIU/mL; 95% CI: −0.86, 0.02 μIU/mL; *P* = 0.06; *I*^2^ = 42%), although it showed a trend of decrease. Subgroup analyses suggested that non-ad libitum TRE combined with exercise resulted in a reduction in fasting insulin (MD: −0.61 μIU/mL; 95% CI: −0.88, −0.34 μIU/mL; *P* < 0.01; *I*^2^ = 1%) but not for the ad libitum TRE group. Subgroup analyses based on intervention duration showed that duration time >4 wk of the combined strategy may reduce fasting insulin concentration (MD: −0.44 μIU/mL; 95% CI: −0.86, −0.02 μIU/mL; *P* = 0.04; *I*^2^ = 43%) but not for the short-term interventions of ≤4 wk compared with a control diet with exercise.

#### Effects on lipid profile

**FIGURE 7:**
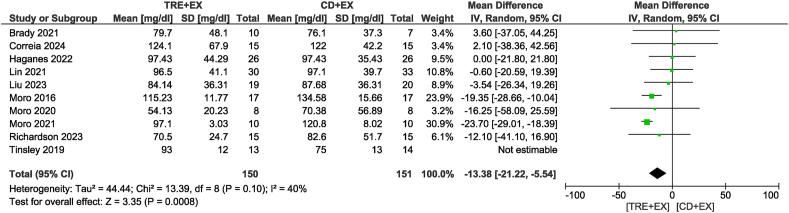
Forest plot of the effects of TRE + EX compared with CD + EX on triglycerides. CD, control diet; CI, confidence interval; EX, exercise; IV, inverse variance; SD, standard deviation; TRE, time-restricted eating.

**FIGURE 8:**
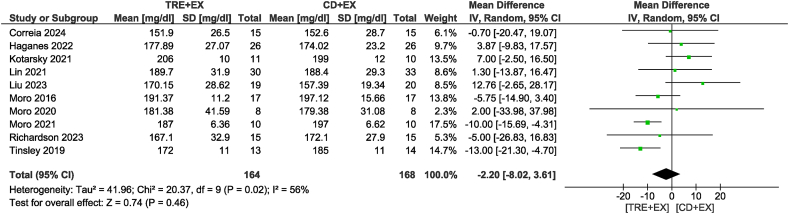
Forest plot of the effects of TRE + EX compared with CD + EX on total cholesterol. CD, control diet; CI, confidence interval; EX, exercise; IV, inverse variance; SD, standard deviation; TRE, time-restricted eating.

**FIGURE 9:**
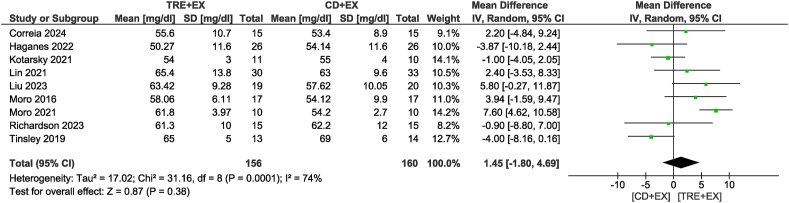
Forest plot of the effects of TRE + EX compared with CD + EX on HDL cholesterol. CD, control diet; CI, confidence interval; EX, exercise; HDL, high-density lipoprotein; IV, inverse variance; SD, standard deviation; TRE, time-restricted eating.

**FIGURE 10:**
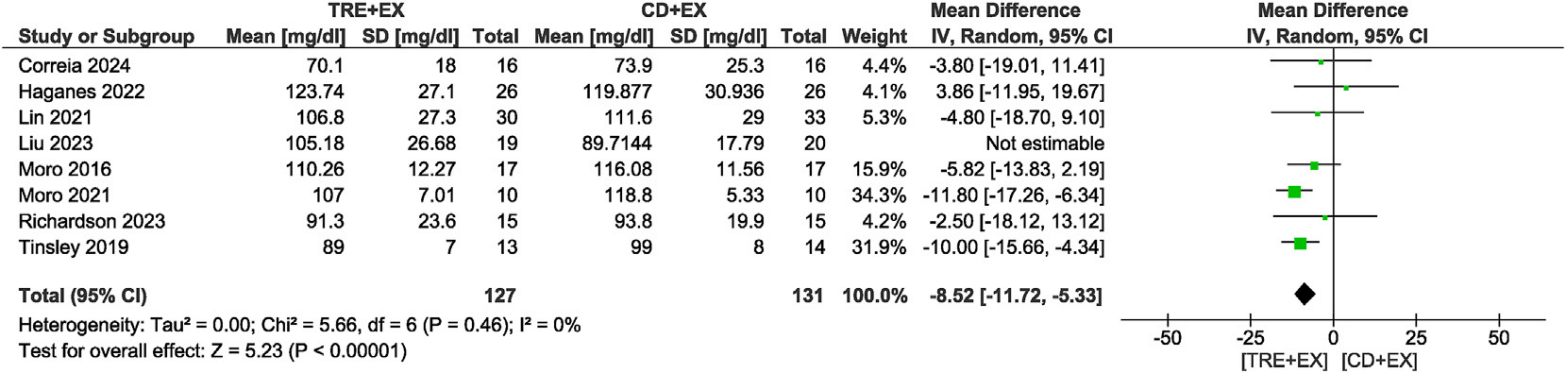
Forest plot of the effects of TRE + EX compared with CD + EX on LDL cholesterol. CD, control diet; CI, confidence interval; EX, exercise; IV, inverse variance; LDL, low-density lipoprotein; SD, standard deviation; TRE, time-restricted eating.

Ten studies [11,12,28,31,33,34,36,37,39,42] were included in the TG analysis, with 328 participants evaluated. There was no difference in TG concentrations between groups (MD: −5.65 mg/dL; 95% CI: −18.93, 7.64 mg/dL; *P* = 0.40; *I*^2^ = 86%) which showed moderate certainty of evidence. Removing the study by Tinsley et al. [42] not only altered statistical significance but also reduced heterogeneity (MD: −13.38 mg/dL; 95% CI: −21.22, −5.54 mg/dL; *P* < 0.01; *I*^2^ = 40%) (Figure 7). For the subgroup analyses, the combined strategy in the non-ad libitum TRE group (MD: −19.23 mg/dL; 95% CI: −26.06, −12.40 mg/dL; *P* < 0.01; *I*^2^ = 27%) and intervention duration time >4 wk group (MD: −12.95 mg/dL; 95% CI: −22.34, −3.55 mg/dL; *P* < 0.01; *I*^2^ = 58%) likely resulted in a reduction in TG concentrations. Ten studies [11,12,[31], [32], [33], [34],^36^,^37^,^39^,^42^], including 332 participants, analyzed TC as an outcome (Figure 8). There was no difference in TC concentrations between groups with very low certainty of evidence (MD: −2.20 mg/dL; 95% CI: −8.02, 3.61 mg/dL; *P* = 0.46; *I*^2^ = 56%). In sensitivity analyses, the elimination of the heterogeneity without changes in statistical significance was observed when removing the study by Kotarsky et al. [32]. Subgroup analysis based on the type of TRE calorie intake revealed significant group differences (*P* < 0.01). Specifically, the non-ad libitum TRE group may have had little to no effect on TC concentrations, but the evidence was very uncertain (MD: −8.82 mg/dL; 95% CI: −12.76, −4.89 mg/dL; *P* < 0.01; *I*^2^ = 0%), whereas no reduction was observed in the ad libitum TRE group. Regarding the analysis of different intervention durations, both duration groups did not show a statistically significant reduction in TC concentrations. In the analysis of HDL, 9 studies [11,12,[31], [32], [33], [34],^36^,^39^,^42^] involving 316 participants were included (Figure 9). The results indicated no significant difference in HDL concentrations between the combined strategy and the control group with very low certainty of evidence (MD: 1.45 mg/dL; 95% CI: −1.80, 4.69 mg/dL; *P* = 0.38; *I*^2^ = 74%). Removing the study by Moro et al. [12] did not alter statistical significance but eliminated heterogeneity. Subgroup analysis also did not reveal any significant differences. Eight studies [11,12,31,33,34,36,39,42] with 297 participants analyzed LDL as an outcome. There was no difference in LDL concentrations between groups with low certainty of evidence (MD: −4.61 mg/dL; 95% CI: −10.01, 0.78 mg/dL; *P* = 0.09; *I*^2^ = 56%). Removing the study by Liu et al. [34] not only altered statistical significance but also reduced heterogeneity (MD: −8.52 mg/dL; 95% CI: −11.72, −5.33 mg/dL; *P* < 0.01; *I*^2^ = 0%) (Figure 10). For the subgroup analyses, the non-ad libitum TRE group (MD: −9.3 mg/dL; 95% CI: −12.65, −5.96 mg/dL; *P* < 0.01; *I*^2^ = 0%) and duration time >4 wk group (MD: −8.74 mg/dL; 95% CI: −12.48, −4.99 mg/dL; *P* < 0.01; *I*^2^ = 14%) may result in a reduction in LDL concentration.

#### Effects on inflammatory cytokines and adipokines

**FIGURE 11:**
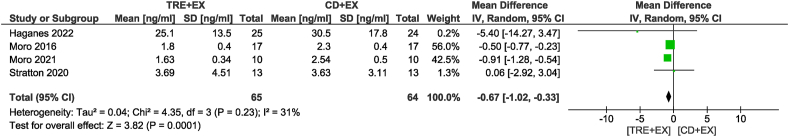
Forest plot of the effects of TRE + EX compared with CD + EX on leptin. CD, control diet; CI, confidence interval; EX, exercise; IV, inverse variance; SD, standard deviation; TRE, time-restricted eating.

**FIGURE 12:**
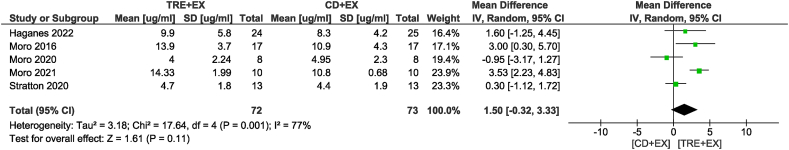
Forest plot of the effects of TRE + EX compared with CD + EX on adiponectin. CD, control diet; CI, confidence interval; EX, exercise; IV, inverse variance; SD, standard deviation; TRE, time-restricted eating.

**FIGURE 13:**
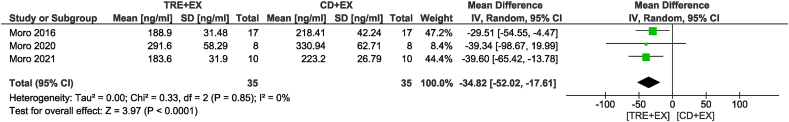
Forest plot of the effects of TRE + EX compared with CD + EX on IGF-1. CD, control diet; CI, confidence interval; EX, exercise; IGF-1, insulin-like growth factor 1; IV, inverse variance; SD, standard deviation; TRE, time-restricted eating.

**FIGURE 14:**
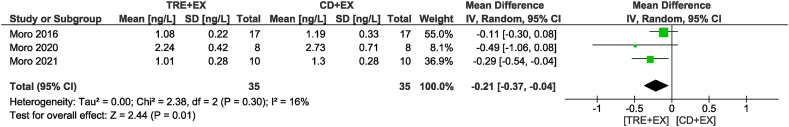
Forest plot of the effects of TRE + EX compared with CD + EX on IL-6. CD, control diet; CI, confidence interval; EX, exercise; IL-6, interleukin 6; IV, inverse variance; SD, standard deviation; TRE, time-restricted eating.

**FIGURE 15:**
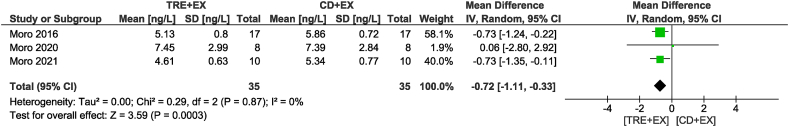
Forest plot of the effects of TRE + EX compared with CD + EX on TNF-α. CD, control diet; CI, confidence interval; EX, exercise; IV, inverse variance; SD, standard deviation; TNF-α, tumor necrosis factor alpha; TRE, time-restricted eating.

Four studies [11,12,36,40] with 129 participants analyzed the leptin value (Figure 11). Participants who submitted to the combined strategy may have experienced a large reduction in leptin value when compared with the control group with low certainty of evidence (MD: −0.67 ng/mL; 95% CI: −1.02, −0.33 ng/mL; *P* < 0.01; *I*^2^ = 31%). Five studies [11,12,36,37,40] with 145 participants analyzed the adiponectin value (Figure 12). Participants who submitted to the combined strategy showed no significant change in adiponectin concentrations compared with the control group with very low certainty of evidence (MD: 1.50 μg/mL; 95% CI: −0.32, 3.33 μg/mL; *P* = 0.11; *I*^2^ = 77%). Three studies [12,36,37] analyzed IGF-1 as an outcome, with 70 participants (Figure 13). The combined strategy may have resulted in a large reduction in IGF-1 concentrations compared with the control group, with low certainty of evidence (MD: −34.82 ng/mL; 95% CI: −52.02, −17.61 ng/mL; *P* < 0.01; *I*^2^ = 0%). Three studies [12,36,37] were included to analyze the IL-6 value with 70 participants (Figure 14). The combined strategy may have resulted in a large reduction in IL-6 concentrations compared with the control group, which showed low certainty of evidence (MD: −0.21 ng/L; 95% CI: −0.37, −0.04 ng/L; *P* = 0.01; *I*^2^ = 16%). Three studies [12,36,37] evaluated TNF-α as an outcome with 70 participants (Figure 15). Participants who submitted to the combined strategy may have experienced in a large decrease in TNF-α concentrations compared with the control group with low certainty of evidence (MD: −0.72 ng/L; 95% CI: −1.11, −0.33; *P* < 0.01; *I*^2^ = 0%).

### Overview of the main findings

The main findings of the meta-analysis are summarized in the section above. The results of the subgroup analysis are listed in Supplementary Tables 2.1 and 2.2 for the TRE calorie intake and intervention duration subgroup, respectively. The tables include MDs with the corresponding 95% CIs for all subgroups separately. Significant group differences were found in TG and TC concentrations (*P* = 0.02 and *P* < 0.01, respectively) for the TRE calorie intake subgroup analysis. For the subgroup analysis of intervention duration, no group differences were found.

### Risk of bias and overall certainty of evidence

The risk of bias for the selected studies is provided in Table 2. Two studies were reported as low risk of bias [38,42], whereas 17 studies had some concerns [11,12,[27], [28], [29], [30], [31], [32], [33], [34], [35], [36], [37],[39], [40], [41], 43], and none of the studies had a high risk of bias. The risk of bias was some concerns in 12 studies for lack of information about the allocation concealment under the domain “bias from randomization process” [[29], [30], [31], [32],[34], [35], [36], [37],[39], [40], [41],^43^], in 17 studies for lack of information under the domain of the deviations from intended interventions [11,12,[27], [28], [29], [30], [31], [32], [33], [34], [35], [36], [37],[39], [40], [41],^43^], and in 6 studies for the bias in the selection of the reported result [12,33,34,36,40,41]. In the 6 crossover design studies that were assessed using the version of RoB 2 for crossover trials [[29], [30], [31],^35^,^39^,^43^], additional consideration regarding bias arising from period and carryover was assessed, and 1 study had some concerns under this domain [29]. The overall certainty of the evidence was assessed using the GRADE tool, which is presented in Table 3. Among the 14 outcomes analyzed, adiponectin, HDL, and TC were classified as very low quality; fat-free mass, fasting glucose, fasting insulin, IGF-1, IL-6, LDL, leptin, and TNF-α were classified as low quality; and body mass, fat mass, and TG were classified as moderate quality. The risk of bias was a serious issue for all 14 analyses. Inconsistency was serious for 3 analyses. Indirectness was not an issue for any analysis. Imprecision was a serious issue for 11 analyses.

**TABLE 2:** Risk of bias assessment

a. Randomized controlled trial						
Study	Randomization process	Deviations from the intended interventions	Missing outcome data	Measurement of the outcome	Selection of the reported result	Overall bias
Batitucci et al., 2022 [27]	Some concerns	Some concerns	Low	Low	Low	Some concerns
Brady et al., 2021 [28]	Low	Some concerns	Low	Low	Low	Some concerns
Haganes et al., 2022 [11]	Low	Some concerns	Low	Low	Low	Some concerns
Kotarsky et al., 2021 [32]	Some concerns	Some concerns	Low	Low	Low	Some concerns
Lin et al., 2022 [33]	Some concerns	Some concerns	Low	Low	Some concerns	Some concerns
Liu et al., 2023 [34]	Some concerns	Some concerns	Low	Low	Some concerns	Some concerns
Moro et al., 2016 [36]	Some concerns	Some concerns	Low	Low	Some concerns	Some concerns
Moro et al., 2020 [37]	Some concerns	Some concerns	Low	Low	Low	Some concerns
Moro et al., 2021 [12]	Low	Some concerns	Low	Low	Some concerns	Some concerns
Peeke et al., 2021 [38]	Low	Low	Low	Low	Low	Low
Stratton et al., 2020 [40]	Some concerns	Some concerns	Low	Low	Some concerns	Some concerns
Tinsley et al., 2017 [41]	Some concerns	Some concerns	Low	Low	Some concerns	Some concerns
Tinsley et al., 2019 [42]	Low	Low	Low	Low	Low	Low

b. Randomized crossover study							
Study	Randomization process	Bias arising from period and carryover effects	Deviations from intended interventions	Missing outcome data	Measurement of the outcome	Selection of the reported result	Overall bias
Correia et al., 2021 [29]	Some concerns	Some concerns	Some concerns	Low	Low	Low	Some concerns
Correia et al., 2023 [30]	Some concerns	Low	Some concerns	Low	Low	Low	Some concerns
Correia et al., 2024 [31]	Some concerns	Low	Some concerns	Low	Low	Low	Some concerns
Martínez-Rodríguez et al., 2021 [35]	Some concerns	Low	Some concerns	Low	Low	Low	Some concerns
Richardson et al., 2023 [39]	Some concerns	Low	Some concerns	Low	Low	Low	Some concerns
Tovar et al., 2021 [43]	Some concerns	Low	Some concerns	Low	Low	Low	Some concerns

**TABLE 3:** Overall certainty of evidence

Certainty assessment							No. of patients		Effect		Certainty	Importance
No. of studies	Study design	Risk of bias	Inconsistency	Indirectness	Imprecision	Other considerations	Time-restricted eating combined with exercise	Control diet combined with exercise	Relative (95% CI)	Absolute (95% CI)		
Body mass												
18	Randomized trials	Serious^1^	Not serious	Not serious	Not serious	None	287	281	—	MD 1.86 kg lower (2.75 lower to 0.97 lower)	⊕⊕⊕◯ Moderate	—
Fat mass												
14	Randomized trials	Serious^1^	Not serious	Not serious	Not serious	none	193	187	—	MD 1.52 kg lower (2.07 lower to 0.97 lower)	⊕⊕⊕◯ Moderate	—
Fat-free mass												
13	Randomized trials	Serious^1^	Not serious	Not serious	Serious^2^	None	200	199	—	MD 0.34 kg lower (0.89 lower to 0.21 higher)	⊕⊕◯◯ Low	—
Adiponectin												
5	Randomized trials	Serious^1^	Serious^3^	Not serious	Serious^2^^,^^4^	None	72	73	—	MD 1.5 μg/mL lower (0.32 lower to 3.33 higher)	⊕◯◯◯ Very low	—
Fasting glucose												
9	Randomized trials	Serious^1^	Not serious	Not serious	Serious^2^	None	165	165	—	MD 0.41 mg/dL lower (2.04 lower to 1.22 higher)	⊕⊕◯◯ Low	—
Fasting insulin												
9	Randomized trials	Serious^1^	Not serious	Not serious	Serious^2^^,^^4^	None	140	139	—	MD 0.42 μlU/mL lower (0.86 lower to 0.02 higher)	⊕⊕◯◯ Low	—
HDL												
9	Randomized trials	Serious^1^	Serious^3^	Not serious	Serious^2^	None	156	160	—	MD 1.45 mg/dL higher (1.8 lower to 4.69 higher)	⊕◯◯◯ Very low	—
IGF-1												
3	Randomized trials	Serious^1^	Not serious	Not serious	Serious^4^	None	35	35	—	MD 34.82 ng/mL lower (52.02 lower to 17.61 lower)	⊕⊕◯◯ Low	—
IL-6												
3	Randomized trials	Serious^1^	Not serious	Not serious	Serious^4^	None	35	35	—	MD 0.21 ng/L lower (0.37 lower to 0.04 lower)	⊕⊕◯◯ Low	—
LDL												
7	Randomized trials	Serious^1^	Not serious	Not serious	Serious^4^	None	127	131	—	MD 8.52 mg/dL lower (11.72 lower to 5.33 lower)	⊕⊕◯◯ Low	—
Leptin												
4	Randomized trials	Serious^1^	Not serious	Not serious	Serious^4^	None	65	64	—	MD 0.67 ng/mL lower (1.02 lower to 0.33 lower)	⊕⊕◯◯ Low	—
TC												
10	Randomized trials	Serious^1^	Serious^c^	Not serious	Serious^2^	None	164	168	—	MD 2.2 mg/dL lower (8.02 lower to 3.61 higher)	⊕◯◯◯ Very low	—
TG												
9	Randomized trials	Serious^1^	Not serious	Not serious	Not serious	None	150	151	—	MD 13.38 mg/dL lower (21.22 lower to 5.54 lower)	⊕⊕⊕◯ Moderate	—
TNF-α												
3	Randomized trials	Serious^1^	Not serious	Not serious	Serious^4^	None	35	35	—	MD 0.72 ng/L lower (1.11 lower to 0.33 lower)	⊕⊕◯◯ Low	—

Abbreviations: CI, confidence interval; HDL, high-density lipoprotein; IGF-1, insulin-like growth factor 1; IL-6, interleukin 6; LDL, low-density lipoprotein; MD, mean difference, TC, total cholesterol; TG, triglyceride; TNF-α, tumor necrosis factor alpha.

1 Most of the included studies exhibited some concerns in the risk of bias assessment, which may have an impact on the certainty of the findings.

2 Imprecise due to confidence intervals included potential for important harm or benefit.

3 *I*^2^ values showed high heterogeneity.

4 Small sample size.

### Publication bias

We assessed publication bias to evaluate the potential impact of selective publication on the results of our meta-analysis. The variables body mass, fat mass, fat-free mass, and TC fulfilled the minimum requirement of including ≥10 studies, enabling a publication bias assessment (Supplementary Table 3). The Egger’s linear regression test for funnel plot asymmetry was used to investigate publication bias, and funnel plots showed no indication of publication bias in body mass (0.532), fat mass (0.06), fat-free mass (0.097), and TC (0.10).

### Future studies

During a search of clinicaltrials.gov, 14 registries of clinical trials were identified, with the combination of TME and exercise as an intervention in different individuals. These trials are ongoing or expected to be completed between 2023 and 2027. Among these studies, different outcomes are being analyzed: body composition (*n* = 14), glycemic profile (*n* = 11), lipid profile (*n* = 10), and inflammatory markers (*n* = 4), as shown in Supplementary Table 4.

## Discussion

Overall, this systematic review and meta-analysis investigated the combined effect of TRE and exercise on body composition and metabolic health in adults compared with a control diet and exercise. Outcome measures included body mass, fat mass, fat-free mass, fasting glucose and insulin, TG, TC, LDL, HDL, leptin, adiponectin, IGF-1, IL-6, and TNF-α. Two studies were categorized as having a low risk of bias [38,42], whereas the other 17 studies raised some concerns [11,12,[27], [28], [29], [30], [31], [32], [33], [34], [35], [36], [37],[39], [40], [41],^43^]. The GRADE evaluation rated 3 of the 14 outcomes from the current study as moderate quality, and the remaining 11 were classified as low and very low quality. The main results of our meta-analysis highlighted the additive effect of TRE in the presence of exercise on body composition and metabolic health, compared with exercise alone. The pooled results from the 19 articles involving 568 participants revealed that TRE plus exercise reduces body mass, fat mass, TG, LDL, leptin, IGF-1, IL-6, and TNF-α. However, no changes were observed in fat-free mass, fasting glucose, insulin, TC, HDL, or adiponectin concentrations. These findings suggest that combining TRE with exercise may effectively improve body composition and specific metabolic markers in adults.

The pooled analysis demonstrated a notable and superior effect of combining TRE with exercise in terms of reducing body mass (−1.86 kg, *P* < 0.01) and fat mass (−1.52 kg, *P* < 0.01) when compared with the control group. These findings are consistent with a previous meta-analysis that showed a significant difference in body weight between IF combined with exercise and the exercise-only groups [22]. Notably, the additional reduction in body mass observed in the combined intervention did not lead to a further decrease in fat-free mass (*P* = 0.23). Preserving fat-free mass during weight loss is crucial due to its role in regulating metabolic rate, maintaining skeletal integrity, and preserving functional capacity [44]. This outcome also aligns with another meta-analysis that reported similar preservation of fat-free mass when implementing IF and resistance training to reduce body mass and body fat [45]. Additionally, a systematic review combining resistance training and IF demonstrated preserved muscle mass and decreased body fat percentage [46]. It is worth noting that TRE restricts the time available for calorie consumption, potentially resulting in an overall decrease in energy intake [47]. During fasting, the body’s stored glycogen reserves become depleted, and it starts to rely more on fat as a fuel source to meet its energy needs. This shift from using predominantly carbohydrates to using more fats for energy is known as increased fat oxidation [48]. On the other hand, exercise promotes energy expenditure and stimulates fat oxidation. The synergistic effects of these interventions likely contributed to the observed improvements in body composition compared with individual interventions. The increased fat mass loss observed in the combined intervention may be attributed to enhanced fat oxidation and a favorable metabolic shift toward an increased oxidative phenomenon [49]. Further research is needed to explore the long-term effects and underlying mechanisms of this combined intervention.

In terms of the glycemic profile, the current meta-analysis revealed that the combination of TRE with exercise did not yield additional benefits on fasting glucose concentrations compared with the control group. However, notable trends were observed, particularly in the non-ad libitum and the longer-duration subgroups, where reductions in fasting insulin concentrations reached statistical significance. This decrease in the insulin concentration may indicate an improvement in insulin resistance, as assessed by HOMA-IR, which takes into account fasting insulin and glucose values [50]. A recent review encompassing studies involving TRE in adults with obesity reported no changes in fasting glucose concentrations after 2–12 mo of TRE [51]. It is worth noting that the studies reporting reductions in fasting insulin concentrations mostly involved TRE interventions with shorter eating windows. Only one systematic review and meta-analysis have examined the combined effects of IF and exercise on glycemic markers compared with exercise alone with a control diet, and it also found no significantly greater improvements in glycemic markers, which aligns with our findings [52]. The variability observed in the effects of combined TRE and exercise on the glycemic profile across different studies can be attributed to several factors. Participant characteristics play a crucial role, as some studies focused on healthy adults with normal glycemic profiles, whereas others targeted individuals with pre-existing metabolic disorders. Participants’ baseline fasting glucose and insulin concentrations can significantly influence the outcomes. Additionally, differences in the design of TRE interventions, such as the duration of fasting, timing of meals, and even the composition of meals consumed before blood samples were taken, can also contribute to variations in the effects on glucose metabolism. It is important to note that TRE differs from a ketogenic or low-calorie diet, because it does not strictly limit carbohydrate intake, thereby minimizing the direct impact on blood glucose concentrations.

The effects of combining TRE with exercise on lipid profiles remain uncertain. Our analysis revealed that the implemented interventions did not impact TC and HDL concentrations. However, there was a notable decrease in LDL concentrations (8.52 mg/dL, *P* < 0.01) and TG concentrations (13.38 mg/dL, *P* < 0.01). These findings are consistent with a previous meta-analysis by Kazeminasab et al. [22], which reported a significant reduction of 5.35 mg/dL in LDL concentrations and a trend toward a reduction in TG when combining IF with exercise compared with a control diet plus exercise. Another umbrella review also highlighted the effect of TRE on LDL concentrations [53]. These observations may be attributed to the influence of TRE on lipid metabolism [54]. TRE has been linked to improved lipolysis and β-oxidation [55]. The fasting state reduces the production of apolipoprotein B in liver cells, lowering LDL concentrations [56]. Additionally, studies have demonstrated that TRE can impact the expression of genes involved in lipid metabolism [57]. Studies in rodent models have shown that time-restricted feeding combined with endurance exercise can enhance fatty acid metabolism and prevent diet-induced fat mass gain [58]. The objective of TRE is to maximize lipid consumption through more extended fasting periods while promoting anabolic processes and preserving lean mass [32], which aligns with our previous findings on body composition. However, other meta-analyses have reported contradictory results. Khalafi et al. [52] found no significant differences in changes to lipid profiles between exercise plus IF and exercise alone. Possible reasons for the discrepancies include variations in intervention types and study designs. These meta-analyses encompassed different forms of IF, including alternate-day fasting, 5:2 IF, TRE, and Ramadan IF, a religious form. It is an obligatory practice to refrain from eating and drinking during daylight hours daily for 29 or 39 d annually [59]. Furthermore, regular exercise promotes favorable changes in lipid metabolism, such as increased lipoprotein lipase activity, which aids in TG breakdown and LDL particle clearance from the bloodstream [60]. The inconsistent findings may be due to ceiling effects regarding the magnitude of improvement. Most participants in our study did not have metabolic disorders and were within a healthy range at baseline. Therefore, the combined intervention may yield more benefits for lipid profiles in individuals with cardiometabolic disorders. Further research is necessary to ascertain whether the combined intervention of TRE and exercise provides greater lipid profile improvements than individual interventions in people with dyslipidemia.

Regarding adipokines, a recent systematic review and meta-analysis examining the effects of TRE on these markers found that TRE improved leptin concentrations but did not increase adiponectin concentrations, which is consistent with our findings [19]. Leptin and adiponectin are important adipokines produced by adipose tissue that play a role in maintaining whole-body metabolism. Adiponectin is associated with improved insulin sensitivity and possesses anti-inflammatory properties [61]. On the other hand, increasing evidence shows that leptin modulates immune responses by increasing the secretion of various cytokines [54] and promoting immune cell activation, proliferation, and chemotaxis [62]. Furthermore, the circulation concentrations of adipokines are influenced by fat mass [19,63,64]. Therefore, reductions in weight and fat mass may lead to concurrent decreases in leptin concentrations and inflammation. In terms of inflammatory cytokines, there is promising evidence suggesting a large reduction in IL-6 and TNF- α concentrations following the combined intervention of TRE and exercise. IL-6 and TNF-α are proinflammatory cytokines associated with chronic inflammation and metabolic dysfunction [65]. TRE and exercise have independently demonstrated anti-inflammatory effects [18,66]. However, it is essential to note that only 3 studies included in this systematic review and meta-analysis measured IL-6 and TNF-α concentrations. Further well-designed studies with larger sample sizes are needed to determine the effectiveness of the combined intervention of TRE and exercise on inflammatory biomarkers.

Another highlighted aspect of the present study was the subgroup analyses performed for different types of TRE based on calorie intake and intervention duration. TRE protocols are often differentiated into “ad libitum” and “non-ad libitum”. Ad libitum TRE allows individuals to consume any food and does not impose calorie restrictions within the designated eating window. In contrast, non-ad libitum TRE involves specific guidelines or restrictions on food choices or calorie intake during the eating window. Our subgroup analysis revealed significant differences in TG concentrations (*P* = 0.02) and TC concentrations (*P* < 0.01) between the ad libitum and non-ad libitum TRE approaches. Notably, only the non-ad libitum TRE approach demonstrated improvements. In this subgroup, studies implemented TRE with either calorie restriction [33] or isocaloric eating [12,36,37,39,42], whereas the control diet followed either the same calorie restriction [33] or isocaloric eating [12,36,37,39,42] pattern without a limited eating window. These findings highlight the importance of considering different TRE approaches’ specific characteristics and guidelines, suggesting that the effect of TRE combined with exercise on TC and TG concentrations varied depending on the type of calorie intake employed. However, it is important to note that these conclusions are based on the available evidence, and further research is needed to confirm and expand on these findings. Particularly, future studies should aim for greater standardization in control diet interventions to enable direct comparisons and establish more convincing results.

The findings of this meta-analysis have important implications for understanding the potential benefits of combined interventions in improving metabolic health and body composition. By synthesizing data from 19 studies, this comprehensive meta-analysis thoroughly evaluates the available evidence. The results indicate that the combined intervention of TRE and exercise yields more substantial changes compared with a control diet with exercise. This suggests a potential synergistic effect, supporting the notion that simultaneously targeting dietary patterns and exercise may have additive or interactive benefits in enhancing body composition and metabolic health. Remarkably, our analysis highlights that the non-ad libitum TRE pattern is associated with more pronounced effects in lipid profiles. However, it is important to acknowledge certain limitations of our meta-analysis. The majority of the included studies focused on healthy and physically active individuals with normal body weights and optimized metabolic parameters. Only 6 studies [11,27,[32], [33], [34],^38^] specifically investigated the combined effect of TRE and exercise in participants with overweight/obesity who may present with abnormal metabolic parameters. Moreover, there was considerable variability in the types of exercise employed across the studies, including resistance training, endurance training, concurrent training, etc. This heterogeneity in exercise types and populations may have influenced the interpretation of the results. To address these limitations, future studies should consider comparing the effects of different exercise modalities and exploring the combined intervention in distinct population groups separately. Another limitation of our meta-analysis is the language restriction to studies published solely in English. This restriction may introduce bias, as relevant studies published in other languages were excluded from our analysis. In summary, although this meta-analysis provides valuable insights into the combined interventions of TRE and exercise, it is crucial to consider the abovementioned limitations when interpreting the results. Further research encompassing a broader range of participant characteristics, exercise types, and languages will enhance our understanding of the topic and allow for more robust conclusions.

Regarding this topic, future studies presented in our search results included 14 trials investigating the combined effect of TRE and exercise. Although specific publications for these studies have not yet been identified, we believe that they hold the potential to provide valuable insights into the effects discussed in this section. Additionally, these studies may contribute to updating the data of this meta-analysis. Furthermore, we anticipate that this review will be a significant resource for guiding future analyses of TRE and exercise interventions, particularly considering the expected increase in related publications. There is a need for future research to focus on investigating the effects of combined TRE and exercise interventions in diverse populations, encompassing individuals with metabolic disorders or sedentary lifestyles. Longer intervention durations should also be explored to understand the sustained effects on metabolic health better. Furthermore, it is crucial to identify the optimal modalities of TRE and exercise to maximize their potential benefits. Conducting studies in these areas will advance our knowledge and enhance the effectiveness of interventions targeting metabolic health.

In conclusion, our systematic review and meta-analysis provides evidence supporting the effectiveness of combining TRE with exercise in reducing body weight and fat mass, as well as improving lipid profiles. However, further research is needed to investigate the comparing effects of different exercise modalities and explore the combined intervention in distinct population groups separately. These findings have significant implications for healthcare practitioners and public health professionals, offering valuable insights into the combined effects of TRE and exercise. Implementing this integrated approach may benefit individuals aiming to achieve weight loss and improve metabolic well-being.

## Author contributions

The authors’ responsibilities were as follows – ZD, KW, SW: conceived and designed research; ZD, KW, RH, CZ; performed review and meta-analysis; ZD, KW: analyzed data and interpreted the results; ZD: drafted the manuscript; ZD, KW, MM, RH, CZ, EP, SW: edited and revised the manuscript; and all authors: read and approved the final manuscript.

## Conflict of interest

The authors report no conflicts of interest.

## Funding

The authors reported no funding received for this study.

## Data availability

Data described in the manuscript, code book, and analytic code will be made available upon request pending.
