# Supplementary material for: The Effect of Time-Restricted Eating Combined with Exercise on Body Composition and Metabolic Health: A Systematic Review and Meta-Analysis
Source: Adv Nutr. 2024 Jun 17;15(8):100262. doi: 10.1016/j.advnut.2024.100262 (PMC11301358; doi:10.1016/j.advnut.2024.100262)
Supplement: Multimedia component 1 [file mmc1.docx]

**Supplementary materials legend**

**Supplementary Table 1.** Search strategy

**Supplementary Table 2.1**. Subgroup analysis according to the TRE calorie intake

**Supplementary Table 2.2**. Subgroup analysis according to the intervention duration

**Supplementary Table 3**. Publication bias

**Supplementary Table 4**. Future study

**Supplementary Table 1**

Search strategy

| **Database** | **Searches** | **Results** |
| --- | --- | --- |
| Embase | ((time-restricted feeding or time-restricted eating or intermittent fasting or time-restricted fasting or time-restricted diet or time-restricted meal) and (exercise or physical activity or physical exercise or training or fitness or running or cycling)).mp. [mp=title, abstract, heading word, drug trade name, original title, device manufacturer, drug manufacturer, device trade name, keyword heading word, floating subheading word, candidate term word] | 102 |
|  | Limiters - Full Text; Humans |  |
| Pubmed | ((time-restricted feeding or time-restricted eating or intermittent fasting or time-restricted fasting or time-restricted diet or time-restricted meal)) AND ((exercise or physical activity or physical exercise or training or fitness or running or cycling)) | 821 |
|  | Filters - Full text, Humans |  |
| SPORTDiscus | ((time-restricted feeding or time-restricted eating or intermittent fasting or time-restricted fasting or time-restricted diet or time-restricted meal) AND (exercise or physical activity or physical exercise or training or fitness or running or cycling)) | 62 |
|  | Limiters - Full Text |  |
| Web of Science | (ALL=(time-restricted feeding or time-restricted eating or intermittent fasting or time-restricted fasting or time-restricted diet or time-restricted meal)) AND ALL=(exercise or physical activity or physical exercise or training or fitness or running or cycling) | 1820 |
|  | Limiters - N/A |  |
| Cochrane Library | time-restricted feeding or time-restricted eating or intermittent fasting or time-restricted fasting or time-restricted diet or time-restricted meal in Title Abstract Keyword AND exercise or physical activity or physical exercise or training or fitness or running or cycling in Title Abstract Keyword | 267 |
|  | In Trials (Word variations have been searched) |  |

| **Outcome** | **Ad libitum** | **MD** | **95%CI** | **I^2^** | **Non-ad libitum** | **MD** | **95%CI** | **I^2^** | **Subgroup differences** |
| --- | --- | --- | --- | --- | --- | --- | --- | --- | --- |
| Body mass (kg) | 8 | -1.67 | [-3.64, 0.31] | 20% | 10 | -1.91 | [-2.97, -0.86] | 26% | Chi^2^= 0.05 (p=0.83) |
| Fat mass (kg) | 7 | -1.02 | [-2.13, 0.09] | 0% | 7 | -1.68 | [-2.31, -1.05] | 0% | Chi^2^= 1.01 (p=0.32) |
| Fat-free mass (kg) | 6 | -0.71 | [-1.58, 0.16] | 0% | 7 | -0.31 | [-1.36, 0.75] | 27% | Chi^2^= 0.34 (p=0.56) |
| Fasting glucose (mg/dl) | 3 | 1.66 | [-1.24, 4.56] | 0% | 6 | -1.36 | [-3.33, 0.61] | 0% | Chi^2^= 2.85 (p=0.09) |
| Fasting insulin (ulU/ml) | 3 | -0.19 | [-1.25, 0.88] | 43% | 6 | -0.61 | [-0.88, -0.34] | 1% | Chi^2^= 0.56 (p=0.45) |
| TG (mg/dl) | 4 | -0.64 | [-14.45, 13.17] | 0% | 5 | -19.23 | [-26.06, -12.40] | 27% | **Chi^2^= 5.59 (p=0.02)** |
| TC (mg/dl) | 4 | 6.48 | [-0.09, 13.05] | 0% | 6 | -8.82 | [-12.76, -4.89] | 0% | **Chi^2^= 15.53 (p<0.01)** |
| HDL (mg/dl) | 4 | 0.47 | [-3.24, 4.18] | 48% | 5 | 2.01 | [-3.02, 7.04] | 81% | Chi^2^= 0.23 (p=0.63) |
| LDL (mg/dl) | 2 | -0.12 | [-11.08, 10.85] | 0% | 5 | -9.30 | [-12.65, -5.96] | 0% | Chi^2^= 2.47 (p=0.12) |

**Supplementary Table 2.1** Subgroup analysis according to the TRE calorie intake

Note: HDL, high density lipoprotein; LDL, low density lipoprotein; MD, mean differences; TC, total cholesterol; TG, triglycerides; TRE, time-restricted eating

| **Outcome** | **≤ 4 weeks** | **MD** | **95%CI** | **I^2^** | **> 4 weeks** | **MD** | **95%CI** | **I^2^** | **Subgroup differences** |
| --- | --- | --- | --- | --- | --- | --- | --- | --- | --- |
| Body mass (kg) | 6 | -1.76 | [-4.37, 0.84] | 0% | 12 | -1.93 | [-2.99, -0.87] | 35% | Chi^2^= 0.01 (p=0.90) |
| Fat mass (kg) | 6 | -1.62 | [-2.50, -0.73] | 0% | 8 | -1.46 | [-2.15, -0.76] | 0% | Chi^2^= 0.08 (p=0.78) |
| Fat-free mass (kg) | 5 | -0.47 | [-2.03, 1.08] | 0% | 8 | -0.42 | [-1.15, 0.32] | 21% | Chi^2^= 0.00 (p=0.95) |
| Fasting glucose (mg/dl) | 3 | 0.19 | [-2.91, 3.29] | 0% | 6 | -0.56 | [-2.57, 1.46] | 4% | Chi^2^= 0.16 (p=0.69) |
| Fasting insulin (ulU/ml) | 2 | -0.09 | [-3.05, 2.87] | 68% | 7 | -0.44 | [-0.86, -0.02] | 43% | Chi^2^= 0.05 (p=0.82) |
| TG (mg/dl) | 3 | -9.44 | [-29.98, 11.09] | 0% | 6 | -12.95 | [-22.34, -3.55] | 58% | Chi^2^= 0.09 (p=0.76) |
| TC (mg/dl) | 3 | -1.98 | [-15.55, 11.59] | 0% | 7 | -1.96 | [-8.89, 4.97] | 70% | Chi^2^= 0.00 (p=1.00) |
| HDL (mg/dl) | 2 | 0.83 | [-4.43, 6.09] | 0% | 7 | 0.21 | [-2.83, 3.26] | 56% | Chi^2^= 0.04 (p=0.84) |
| LDL (mg/dl) | 2 | -3.17 | [-14.07, 7.73] | 0% | 5 | -8.74 | [-12.48, -4.99] | 14% | Chi^2^= 0.90 (p=0.34) |

**Supplementary Table 2.2** Subgroup analysis according to the intervention duration

Note: HDL, high density lipoprotein; LDL, low density lipoprotein; MD, mean differences; TC, total cholesterol; TG, triglycerides

**Supplementary Table 3** Publication bias result

Body mass P=0.532 Fat mass P=0.06


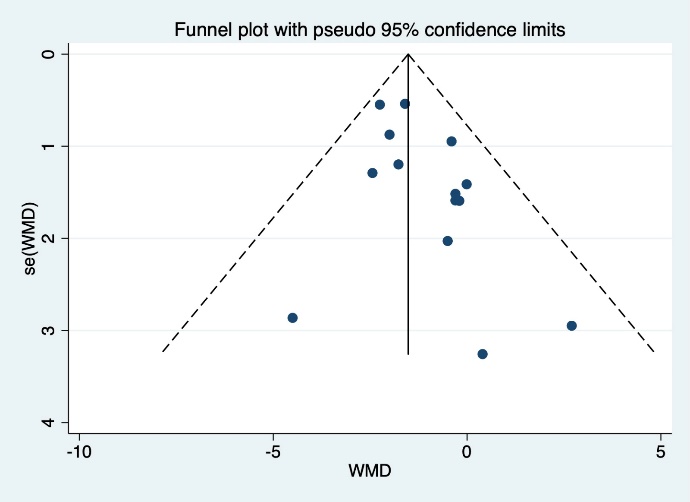

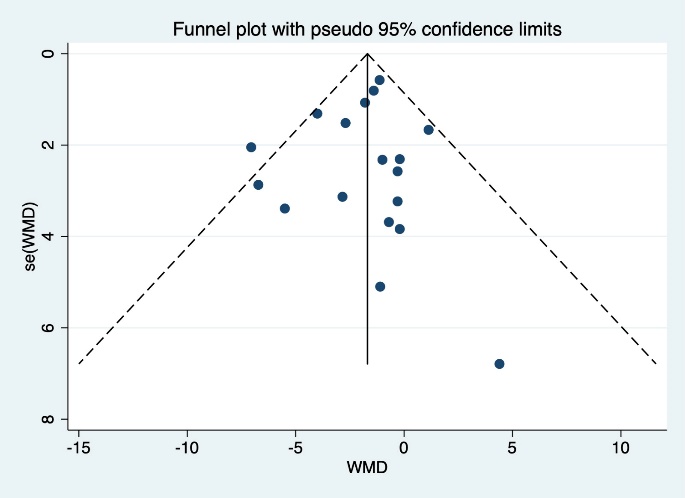


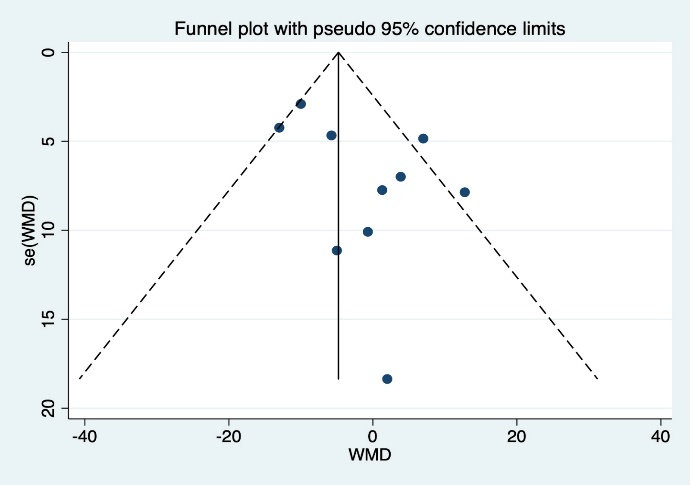

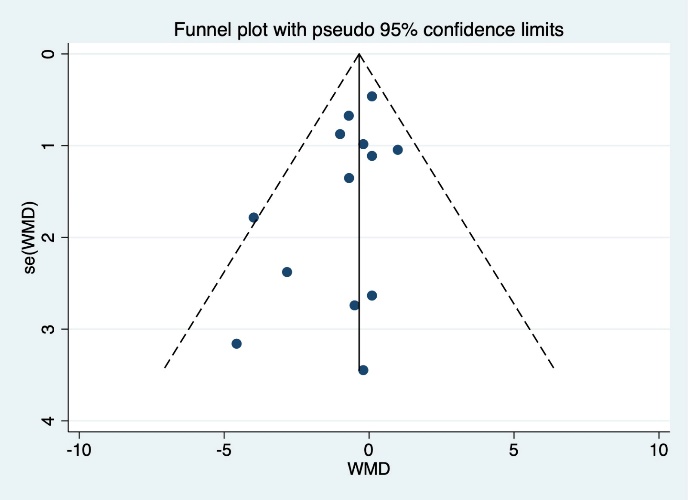
Fat-free mass P=0.097 Total cholesterol P=0.10

| **Supplementary Table 4** Future study Studies registered at clinicaltrials.gov in individuals submitted to time-restricted eating combined with exercise and the presence of body composition, glycemic profile, lipid profile, and inflammatory markers as analyzed outcomes. | | | | | | | |
| --- | --- | --- | --- | --- | --- | --- | --- |
| **Register** | **Study beginning (year)** | **Completion forecast (year)** | **Body composition** | **Glycemic profile** | **Lipid profile** | **Inflammatory markers** | **Population** |
| NCT04978376 | 2021 | 2023 | YES | YES | NO | NO | Overweight and obese |
| NCT05486702 | 2022 | 2023 | YES | NA | NA | NA | Obesity |
| NCT05229835 | 2022 | 2027 | YES | YES | YES | NO | Older women and young men |
| NCT05897073 | 2023 | 2025 | YES | YES | YES | YES | Obesity |
| NCT05908201 | 2023 | 2024 | YES | NO | NO | NO | Resistance trained male |
| NCT05167903 | 2022 | 2023 | YES | YES | YES | NO | Metabolic syndrome |
| NCT05912309 | 2023 | 2025 | YES | YES | YES | NO | Postmenopausal women with obesity |
| NCT05865639 | 2022 | 2023 | YES | YES | YES | YES | Sedentary adults |
| NCT05505305 | 2022 | 2024 | YES | YES | YES | NO | Obesity |
| NCT05290233 | 2023 | 2024 | YES | YES | NO | NO | Obesity and pre-diabetes |
| NCT04834687 | 2021 | 2023 | YES | YES | YES | YES | Overweight students |
| NCT05332613 | 2022 | 2025 | YES | NO | YES | NO | Non-alcoholic fatty liver disease |
| NCT06138015 | 2023 | NA | YES | YES | YES | YES | Menopausal women |
| NCT05996042 | 2023 | NA | YES | YES | YES | NO | Sedentary adults |
